# Magnetic resonance-guided laser interstitial thermal therapy vs. open surgery for drug-resistant mesial temporal lobe epilepsy: a propensity score matched retrospective cohort study

**DOI:** 10.1097/JS9.0000000000000811

**Published:** 2023-10-04

**Authors:** Jiajie Mo, Zhihao Guo, Xiu Wang, Jianguo Zhang, Wenhan Hu, Xiaoqiu Shao, Lin Sang, Zhong Zheng, Chao Zhang, Kai Zhang

**Affiliations:** Departments ofa Neurosurgery; b Neurology, Beijing Tiantan Hospital; c Department of Neurosurgery, Beijing Neurosurgical Institute, Capital Medical University; d China National Clinical Research Center for Neurological Disease, NCRC-ND; e Department of Neurosurgery, Beijing Fengtai Hospital, Beijing, China

**Keywords:** drug-resistant mesial temporal lobe epilepsy, magnetic resonance-guided laser interstitial thermal therapy, open surgery, propensity score matching

## Abstract

**Background:**

Magnetic resonance-guided laser interstitial thermal therapy (MRgLITT) and traditional open surgery (OS) are effective and safe options for patients with drug-resistant mesial temporal lobe epilepsy (DR-mTLE). However, their superiority in seizure control and preservation of functional abilities remains unclear. This study aimed to compare the surgical outcomes of MRgLITT and OS.

**Materials and methods:**

This multicenter retrospective cohort study included patients with DR-mTLE who underwent MRgLITT or OS at three centres between 2015 and 2023. The data on patient demographics, presurgical non-invasive evaluation, stereoelectroencephalography (SEEG) implantation, memory alteration, and seizure outcomes were collected. Propensity score matching (PSM) analysis was conducted for the comparison of seizure control and functional preservation between two surgical approaches.

**Results:**

Of the 244 individuals who met the study criteria, 33 underwent MRgLITT and 211 OS. The median (interquartile range) age at seizure onset was 22.0 (13.0) and 12.3 (10.0) years in the MRgLITT and OS groups, respectively. The first PSM, based on demographic and non-invasive information, resulted in 26 matched pairs for the primary analysis. There were no significant differences in memory preservation (*P* = 0.95) or surgical outcomes (*P* = 0.96) between the groups. The second PSM, based on demographics and SEEG implantation, yielded 32 matched pairs for the sensitivity analysis, showing similar results. Subset analysis of early and late MRgLITT cases revealed no statistically significant differences in the proportion of patients with memory decline (*P* = 0.42) or seizure control (*P* = 1.00). Patients who underwent SEEG implantation were 96% less likely to achieve seizure freedom after MRgLITT (*P* = 0.02).

**Conclusion:**

Minimally invasive MRgLITT is associated with memory preservation and seizure control, similar to traditional OS. MRgLITT is effective and safe for DR-mTLE and is relevant for future prospective randomized trials on dominant-side mTLE, providing practical implications for guiding neurosurgeons in the selection of surgical approaches.

## Graphical abstract

**Figure:**
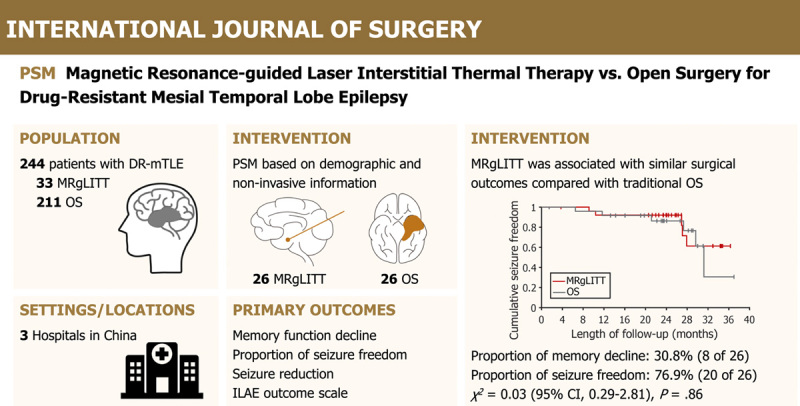

## Introduction

Highlights

- Minimally invasive magnetic resonance-guided laser interstitial thermal therapy (MRgLITT) is associated with memory preservation and seizure control, similar to traditional open surgery.
- The MRgLITT procedure may be relatively simple to use during the learning curve.
- Future prospective randomized trials for MRgLITT on dominant-side mesial temporal lobe epilepsy are expected to provide practical implications for neurosurgeons.

Temporal lobe epilepsy (TLE) represents the most common form of focal epilepsy, accounting for ~66% of the cases^1^. Unfortunately, up to 71% of TLE patients experience drug resistance, making effective treatment challenging^2^. To address this, surgical interventions such as anterior temporal lobectomy (ATL) and selective amygdalohippocampectomy (SAH) have been developed to alleviate seizures and enhance the overall quality of life in individuals with drug-resistant TLE^3–5^. However, open surgery (OS) is an invasive approach with potential risks and complications, including cognitive impairment and visual field deficits^6^.

Advancements in technology have introduced innovative and minimally invasive procedures, such as magnetic resonance-guided laser interstitial thermal therapy (MRgLITT)^7^. MRgLITT employs laser energy guided by real-time MRI to thermally ablate the epileptic focus, enabling precise targeting and monitoring of the ablation process^8,9^. By providing a less invasive alternative to OS, MRgLITT holds the potential to minimize surgical risks, shorten recovery duration, and optimize seizure control in individuals with drug-resistant mesial temporal lobe epilepsy (DR-mTLE)^10^. However, most of previous studies did not include a comparator such as OS and the interpretation of findings was limited by small sample size^11^.

This study aimed to investigate the comparative effectiveness of epilepsy surgery for DR-mTLE by specifically examining the surgical outcomes of MRgLITT versus OS. By elucidating the true benefits of these approaches, we aimed to provide valuable insights into the field of epilepsy surgery and inform decision-making in the management of DR-mTLE.

## Materials and methods

### Data collection

This study was approved by the Institutional Review Board of our Hospital (QX2020-11-02) adhering to the principles of the Declaration of Helsinki and Strengthening the Reporting of Cohort, cross-sectional and case-control Studies in Surgery (STROCSS) guidelines^12^, Supplemental Digital Content 1, http://links.lww.com/JS9/B163 and the protocol for the study was registered at clinicaltrials.gov NCT04569071.

This multicenter retrospective cohort study included patients diagnosed with DR-mTLE who underwent either temporal ablation or lobectomy at three epilepsy centres between January 2015 and May 2023. All patients underwent a standard multidisciplinary presurgical evaluation aimed at addressing the anatomo-electro-clinical profile and determining the surgical strategy. The inclusion criteria were refractory epilepsy (unresponsive to a minimum of three medications for at least 2 years) and presurgical data demonstrating evidence of a unilateral epileptogenic zone (EZ) in the anteromedial temporal region, as assessed through clinical evaluations, scalp video-electroencephalography (EEG), neuropsychological assessments, neuroimaging findings, and, where available, stereoelectroencephalography (SEEG). The exclusion criteria included intracranial space-occupying lesions, dual pathology, incomplete data, and reoperation history.

### Definitions

**Figure 1:**
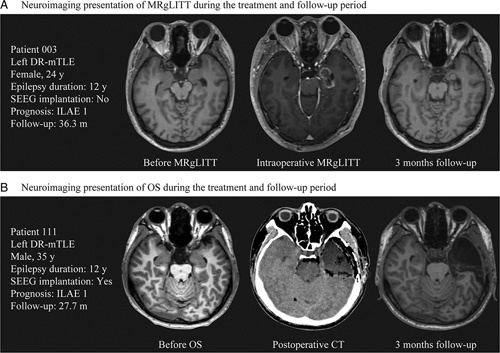
Neuroimaging presentation of minimally invasive magnetic resonance-guided laser interstitial thermal therapy (A) and traditional open surgery (B) in patients with drug-resistant mesial temporal lobe epilepsy. DR-mTLE, drug-resistant mesial temporal lobe epilepsy; ILAE, International League Against Epilepsy outcome scale; MRgLITT, magnetic resonance-guided laser interstitial thermal therapy; OS, open surgery; SEEG, stereoelectroencephalography;.

MRgLITT entails the insertion of a laser fibre through a minor burr hole for ablating mesial temporal structures under real-time MRI temporal monitoring (Fig. 1)^13^. The traditional OS, comprising ATL and SAH, was considered as comparative treatment approach. ATL involves the removal of the anterior portion of the temporal lobe and mesial temporal structures, while SAH involves the resection of the mesial structures, including the hippocampus and amygdala, while sparing the lateral temporal neocortex^14^. Surgical strategy selection was not restricted in the presurgical evaluation procedure. However, MRgLITT was recommended for patients with dominant-side temporal epilepsy because this minimally invasive approach could potentially preserve cognitive and memory functions while retaining the option for subsequent OS if seizures persist. The ultimate procedural determination resulted from thorough discussions between the clinicians and patients.

Seizure control and functional preservation stand as pivotal considerations in epilepsy neurosurgery. Consequently, the assessment of postoperative seizure control prognosis involves metrics such as the rate of seizure freedom, seizure reduction ratio, and International League Against Epilepsy (ILAE) outcome scale^15^. Additionally, the presence of memory impairment serves as an indicator for evaluating functional preservation during the final follow-up.

### Statistical analysis

Continuous data were presented as median (interquartile range), while categorical data were reported as count and percentage. Comparisons between the MRgLITT and OS groups were performed using Student’s *t*-test or Mann–Whitney *U* test for continuous variables and *χ*^*2*^ test or Fisher’s exact test for categorical variables. Symmetric measures were applied to measure the strength of association between two categorical variables: Kendall’s tau-c was used for ordinal data, and Phi for nominal data. The balance of covariates between the treatment groups was assessed using the standardized mean difference (SMD), with an SMD of less than 0.1 indicating an ideal balance.

A 1:1 propensity score matching (PSM) algorithm without replacement, with a caliper of 0.1, was employed to adjust for nonrandom assignment of the procedure. The propensity score was estimated through multivariable logistic regression using the following categories of variables: (1) basic demographic information (sex, lateralization of EZ), (2) EEG findings (interictal and ictal discharge pattern), (3) semiology (aura, loss of awareness, automatism, contralateral dystonia, focal to bilateral tonic-clonic seizures), (4) neuroimaging representations (ipsilateral hippocampal atrophy, incomplete hippocampal inversion, temporal pole atrophy/blurring, hyperintensity of fluid-attenuated inversion recovery, hypometabolism of ^18^F-fluorodeoxyglucose positron emission tomography), and (5) SEEG implantation^16^. Two PSM analyses were conducted: the primary analysis utilized basic demographic and non-invasive information, following the standard protocol for presurgical evaluation in epilepsy surgery; the sensitivity analysis employed the second PSM based on basic demographic and SEEG implantation information. In clinical practice, SEEG provides an objective measure of discordant anatomo-electro-clinical correlations. Kaplan–Meier curve analysis as performed to estimate seizure freedom rates at different time points and to visualize differences in time-to-seizure recurrence between the subgroups. Survival curves between the two cohorts were compared using log-rank (Mantel–Cox) test.

Binary logistic regression was used to identify the independent predictors of seizure freedom following MRgLITT. A forward likelihood ratio (LR) stepwise-selection method was used. The constructed models established an association between seizure freedom and independent variables, encompassing clinical factors such as sex, lateralization, age at seizure onset, duration of epilepsy, and SEEG implantation. To assess the fitness of the model, Hosmer–Lemeshow test was performed, while the omnibus test and computation of Nagelkerke’s *R* squared furnished a comprehensive evaluation of its overall performance. Consequently, the odds ratios (OR), along with their corresponding 95% CI and *P* values, were computed.

Statistical significance was set at a two-sided *P* value of less than 0.05. SPSS (Version 26.0, IBM Corp) software was used for all statistical analyses.

## Results

### Demographic characteristics

**Figure 2:**
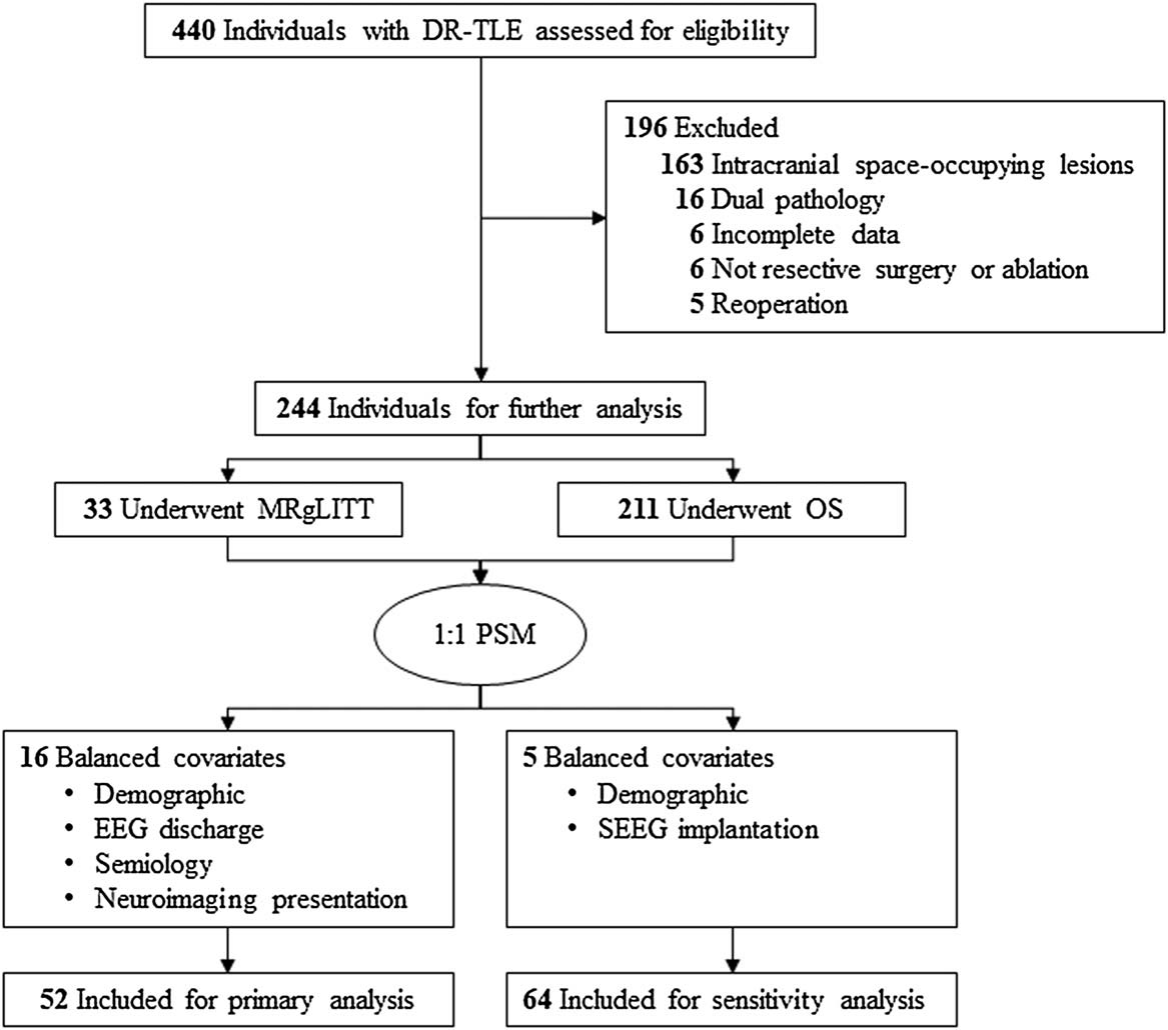
Flowchart of patient recruitment and 1:1 propensity score matching. DR-mTLE, drug-resistant mesial temporal lobe epilepsy; EEG, electroencephalography; MRgLITT, magnetic resonance-guided laser interstitial thermal therapy; OS, open surgery; PSM, propensity score matching; SEEG, stereoelectroencephalography.

A total of 244 patients were eventually recruited for the analysis, with 33 in the MRgLITT group (15 men and 18 women) and 211 in the OS group (109 men and 102 women) (Fig. 2). Table 1, Table 2, SDC Table 1, Supplemental Digital Content 2, http://links.lww.com/JS9/B164, and SDC Table 2, Supplemental Digital Content 2, http://links.lww.com/JS9/B164 show the demographic and clinical characteristics of the study participants. Patients in the MRgLITT group were significantly younger at seizure onset [median (interquartile range), 22.0 (13.0) vs. 12.3 (10.0) years, *P* < 0.001] than those in the OS group. The MRgLITT group had a higher proportion of patients with focal ictal discharge on long-term EEG monitoring [20/33 (60.6%) vs. 79/211 (37.4%), *P* = 0.01], temporal pole atrophy/blurring [20/33 (60.6%) vs. 86/211 (40.8%), *P* = 0.03], and SEEG implantation [8/33 (24.2%) vs. 95/211 (45.0%), *P* = 0.03].

**Table 1:** Comparison between the baseline characteristics of magnetic resonance-guided laser interstitial thermal therapy and open surgery.

	No. (%)											
	Before PSM (*n* = 244)				1^st^ PSM for primary analysis (*n* = 52)				2^nd^ PSM for sensitivity analysis (*n* = 64)			
Characteristic	MRgLITT (*n* = 33)	OS (*n* = 211)	*P*	SMD	MRgLITT (*n* = 26)	OS (*n* = 26)	*P*	SMD	MRgLITT (*n* = 32)	OS (*n* = 32)	*P*	SMD
Sex (male)	15 (45.5)	109 (51.7)	0.51	0.12	13 (50.0)	13 (50.0)	1.00	0	15 (46.9)	15 (46.9)	1.00	0
Laterization (left)	17 (51.5)	101 (47.9)	0.70	0.12	13 (50.0)	14 (53.8)	0.78	0.08	16 (50.0)	16 (50.0)	1.00	0
Age at seizure onset, median (IQR), year	22.0 (13.0)	12.3 (10.0)	< 0.001^*^	1.02	19.5 (12.2)	16.0 (11.5)	0.29	0.30	21.5 (12.8)	17.0 (11.8)	0.25	0.29
Epilepsy duration, median (IQR), year	10.0 (15.0)	14.0 (12.0)	0.29	-0.16	8.5 (15.0)	10.5 (10.8)	0.94	0.10	8.5 (14.5)	11.0 (9.8)	0.71	0.24
Interictal discharge (focal)	16 (48.5)	96 (45.5)	0.75	-0.06	14 (53.8)	14 (53.8)	1.00	0	/	/	/	/
Ictal discharge (focal)	20 (60.6)	79 (37.4)	0.01^*^	-0.48	14 (53.8)	17 (65.4)	0.40	0.24	/	/	/	/
Aura (yes)	25 (75.8)	153 (75.8)	0.70	-0.07	20 (76.9)	20 (76.9)	1.00	0	/	/	/	/
Loss of awareness (yes)	33 (100.0)	197 (93.4)	0.23	-0.29	26 (100.0)	25 (96.2)	1.00	-0.28	/	/	/	/
Automatism (yes)	29 (87.9)	179 (84.8)	0.80	-0.09	23 (88.5)	20 (76.9)	0.47	-0.31	/	/	/	/
Contralateral dystonia (yes)	11 (33.3)	80 (37.9)	0.61	0.10	10 (38.5)	9 (34.6)	0.78	-0.08	/	/	/	/
FBTCS (yes)	16 (48.5)	70 (33.2)	0.09	-0.32	12 (46.2)	14 (53.8)	0.58	0.15	/	/	/	/
Hippocampal atrophy (yes)	24 (72.7)	133 (63.0)	0.28	-0.20	18 (69.2)	18 (69.2)	1.00	0	/	/	/	/
Incomplete hippocampal inversion (yes)	14 (42.4)	77 (36.5)	0.51	-0.12	12 (46.2)	8 (30.8)	0.25	-0.32	/	/	/	/
Temporal pole atrophy/blurring (yes)	20 (60.6)	86 (40.8)	0.03^*^	-0.37	15 (57.7)	16 (61.5)	0.78	0.08	/	/	/	/
Hyperintensity of FLAIR (yes)	32 (97.0)	177 (83.9)	0.06	-0.38	25 (96.2)	25 (96.2)	1.00	0	/	/	/	/
Hypometabolism of ^18^F-FDG PET (yes)	32 (97.0)	201 (95.3)	1.00	-0.08	25 (96.2)	23 (88.5)	0.61	-0.29	/	/	/	/
SEEG implantation (yes)	8 (24.2)	95 (45.0)	0.03^*^	0.43	/	/	/	/	8 (25.0)	11 (34.4)	0.41	0.21

^18^F-FDG PET, ^18^F-fluorodeoxyglucose positron emission tomography; FBTCS, focal to bilateral tonic-clonic seizures; FLAIR, fluid-attenuated inversion recovery; IQR, interquartile range; MRgLITT, magnetic resonance-guided laser interstitial thermal therapy; OS, open surgery; PSM, propensity score matching; SEEG, stereoelectroencephalography; SMD, standardized mean difference.

* Significance.

**Table 2:** Postoperative outcomes according to the type of surgery after propensity score matching.

	No. (%)							
	1^st^ PSM for primary analysis (*n* = 51, 1 patient was lost to follow-up)				2^nd^ PSM for sensitivity analysis (*n* = 61, 3 patients were lost to follow-up)			
Variable	MRgLITT (*n* = 26)	OS (*n* = 25)	*P* value	Strength of association	MRgLITT (*n* = 32)	OS (*n* = 29)	*P* value	Strength of association
Memory function								
Worse	8 (30.8)	11 (44.0)	0.95	Kendall’s tau-c = −0.04, *P* = 0.77	9 (28.1)	10 (34.5)	0.63	Kendall’s tau-c = −0.04, *P* = 0.75
No change	17 (65.4)	10 (40.0)			21 (65.6)	13 (44.8)		
Better	1 (3.8)	4 (16.0)			2 (6.3)	6 (20.7)		
Seizure freedom (yes)	20 (76.9)	19 (76.0)	0.94	Phi = 0.01, *P* = 0.94	23 (71.9)	23 (79.3)	0.50	Phi = −0.09, *P* = 0.50
Seizure frequency reduction, median (IQR)	100.0 (0.0)	100.0 (0.6)	0.86	/	100.0 (2.6)	100.0 (0.0)	0.44	/
ILAE outcome scale								
1	20 (76.9)	19 (76.0)	0.96	Kendall’s tau-c = 0.00, *P* = 1.00	23 (71.9)	23 (79.3)	0.63	Kendall’s tau-c = −0.07, *P* = 0.52
2	1 (3.8)	0			3 (9.4)	0		
3	1 (3.8)	3 (12.0)			1 (3.1)	3 (10.3)		
4	3 (11.5)	3 (12.0)			4 (12.5)	3 (10.3)		
5	1 (3.8)	0			1 (3.1)	0		
6	0	0			0	0		

ILAE, International League Against Epilepsy; IQR, interquartile range; MRgLITT, magnetic resonance-guided laser interstitial thermal therapy; OS, open surgery; PSM, propensity score matching.

### PSM results

The PSM results are shown in Table 1, Table 2, SDC Table 1, Supplemental Digital Content 2, http://links.lww.com/JS9/B164, SDC Table 2, Supplemental Digital Content 2, http://links.lww.com/JS9/B164, and SDC Figure. 1, Supplemental Digital Content 2, http://links.lww.com/JS9/B164).

**Figure 3:**
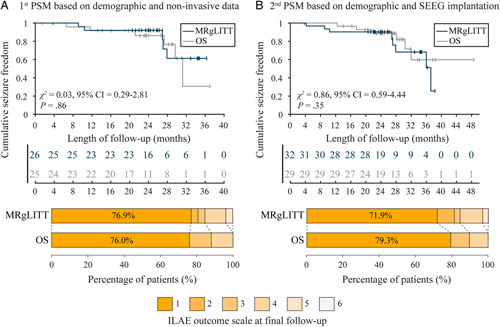
Overall proportion of seizure freedom after propensity score matching. Kaplan–Meier curves with risk tables demonstrate cumulative seizure freedom and the row distribution of International League Against Epilepsy outcome scales over the follow-up period in magnetic resonance-guided laser interstitial thermal therapy and open surgery groups after matching (A) demographic and non-invasive presurgical evaluation information and (B) demographic and stereoelectroencephalography implantation. MRgLITT, magnetic resonance-guided laser interstitial thermal therapy; ILAE, International League Against Epilepsy; OS, open surgery; PSM, propensity score matching; SEEG, stereoelectroencephalography.

The first PSM with a 1:1 ratio yielded 26 matched pairs for primary analysis. Both groups were moderately balanced in all baseline demographic and non-invasive presurgical evaluation information in the matched cohort. The MRgLITT group had fewer opportunities to experience memory decline; however, the result were not significantly different [8/26 (30.8%) vs. 11/25 (44.0%), *P* = 0.95]. These two groups showed no significant difference in seizure freedom [20/26 (76.9%) vs. 19/25 (76.0%), *P* = 0.96] and the trend across the stratified ILAE outcome scale (*P* = 1.00) at the last follow-up. There were no differences in time-to-seizure freedom on Kaplan–Meier analysis between the MRgLITT and OS groups (*χ*^*2*^ = 0.03, 95% CI = 0.29–2.81, *P* = 0.86) (Fig. 3A).

The second PSM yielded 32 matched pairs for sensitivity analysis which matched the baseline demographic and SEEG implantation. The MRgLITT group had a similar functional reservation [9/32 (28.1%) vs. 10/29 (34.5%), *P* = 0.63] and seizure control outcomes [23/32 (71.9%) vs. 23/29 (79.3%), *P* = 0.63] compared to the OS group. The trends across the stratified ILAE outcome scale (*P* = 0.52) at the last follow-up showed no significant difference. The time-to-seizure freedom on Kaplan–Meier analysis when comparing MRgLITT and OS groups (*χ*^*2*^ = 0.86, 95% CI = 0.59–4.44, *P* = 0.35) also did not show any difference (Fig. 3B).

### Comparison of the early- surgery and late-surgery cases that underwent MRgLITT

Furthermore, we analyzed a subset of 33 patients who underwent MRgLITT to reduce the confounding effect of the learning curve. We equally divided them into an early-surgery subset (16 cases, pre50%) and a late-surgery subset (16 cases, post50%) according to the duration of surgery. In the matched cohort, both subsets had no significant difference in the proportion of patients with memory decline [3/16 (18.8%) vs. 6/16 (37.5%), *P* = 0.16] or seizure control [12/16 (75.0) vs. 12/16 (75.0%), *P* = 1.00] (Table 3). There was no statistically significant difference (*χ*^*2*^ = 6.51, 95% CI = 0.04–1.28, *P* = 0.01) in seizure freedom rates between the early-surgery and late-surgery subsets, as depicted in SDC Figure. 2, Supplemental Digital Content 2, http://links.lww.com/JS9/B164.

**Table 3:** Comparison of postoperative outcomes before and after magnetic resonance-guided laser interstitial thermal therapy in 50% of the cases.

	No. (%)			
Variable	Before 50% cases (*n* = 16)	After 50% cases (*n* = 16)	*P*	Strength of association
Memory function				
Worse	3 (18.8)	6 (37.5)	0.16	Kendall’s tau-c = −0.23, *P* = 0.15
No change	12 (75.0)	10 (62.5)		
Better	1 (6.3)	0		
Seizure freedom	13 (81.3)	12 (75.0)	1.00	Phi = 0.09, *P* = 0.67
Seizure frequency reduction, median (IQR)	100.0 (0.0)	100.0 (18.8)	0.80	/
ILAE outcome scale				
1	12 (75.0)	12 (75.0)	1.00	Kendall’s tau-c = 0.00, *P* = 1.00
2	2 (12.5)	1 (6.3)		
3	0	1 (6.3)		
4	1 (6.3)	2 (12.5)		
5	1 (6.3)	0		
6	0	0		

ILAE, International League Against Epilepsy; IQR, interquartile range.

### Evaluating independent predictors of seizure freedom following MRgLITT

The binary logistic regression model showed a good fit (*χ*^*2*^ = 8.07, *P* = 0.43) in the Hosmer–Lemeshow test; *P* = 0.01 in the Omnibus test; Nagelkerke *R* squared = 0.52). Among the clinical variables, patients with SEEG implantation were 96% (95% CI = 0.002–0.63, *P* = 0.02) less likely to achieve seizure freedom following MRgLITT compared with patients who directly underwent epilepsy surgery (SDC Figure. 3, Supplemental Digital Content 2, http://links.lww.com/JS9/B164).

## Discussion

### Summary

A retrospective, multicenter study was performed to compare the outcomes of minimally invasive MRgLITT and traditional OS in patients with DR-mTLE. We found that the MRgLITT had memory preservation and seizure control outcomes comparable to those of OS. Furthermore, after adjusting for the learning curve effect, analysis of the postoperative outcomes in the late-surgery subset of MRgLITT revealed no statistically significant differences compared to the early-surgery subset, indicating similar surgical outcomes between the two groups. This suggests that a minimally invasive approach may help to overcome the learning curve associated with DR-mTLE during the initial phase.

### Interpretations and implications

The limited ablation range of temporal lobectomy remains a hurdle in the development of MRgLITT for DR-mTLE. The laser trajectory passed through the amygdala and hippocampus; hence, the ablation range was similar to the respective range of SAH. Two meta-analyses have reported no significant difference in surgical seizure outcomes and intelligence between ATL and SAH^17,18^. Moreover, a recent meta-analysis revealed that both ATL and SAH are superior to MRgLITT in terms of seizure freedom rates (69% and 66% vs. 57%), and the ablation of mesial hippocampal sclerosis was associated with a significantly higher seizure freedom rate after MRgLITT (64%)^11^. Prior to PSM, a higher prevalence of concordant presurgical evaluation factors was observed in the MRgLITT group, including a focal ictal discharge pattern on long-term monitoring, temporal pole atrophy/blurring, and a decreased likelihood of requiring SEEG implantation. Furthermore, we found that SEEG implantation, signifying patients characterized by contradictory anatomo-electro-clinical correlations based on non-invasive approaches, independently predicted reduced odds of achieving seizure freedom following MRgLITT. Collectively, these findings indicated that MRgLITT can achieve satisfactory outcomes in specific patient populations with focal EZ.

**Figure 4:**
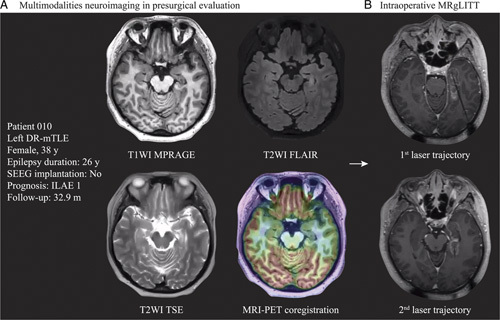
Illustration of two-trajectory approach of magnetic resonance-guided laser interstitial thermal therapy. (A) The multimodalities neuroimaging representations in presurgical evaluation are shown in the left panel. (B) The ablative range of 2 laser trajectories covers the angled geometry relationship of mesial temporal structures. DR-mTLE, drug-resistant mesial temporal lobe epilepsy; MRgLITT, magnetic resonance-guided laser interstitial thermal therapy; MRI-PET co-registration, magnetic resonance imaging-positron emission tomography; SEEG, stereoelectroencephalograph; T1WI MPRAGE, T1-weighted magnetization-prepared rapid gradient echo; T2WI FLAIR, T2-weighted fluid-attenuated inversion recovery; T2WI TSE, T2-weighted turbo spin-echo.

After PSM, both groups exhibited a higher seizure freedom rate than that previously reported (MRgLITT: 76.9% vs. 57%; OS: 76.0% vs. 67.8%)^11^, which may be attributed to the preference for MRgLITT in individuals with a definitive anatomo-electro-clinical correlation during presurgical evaluation. A previous study suggested that the resection of the piriform cortex, rather than other mesiotemporal structures (i.e. hippocampus, amygdala, and entorhinal cortex), is associated with better seizure outcomes^19^. The piriform cortex is located in the superior and inferior banks of the entorhinal sulcus and rostromedially positioned to the amygdala^20^. The complex geometry of the piriform-hippocampus-amygdala-entorhinal region, which is not a typical cylindrical shape, makes it challenging to target with a single laser trajectory and achieve adequate ablation, particularly when considering optic nerve and brainstem avoidance. This challenge has led to the development of a novel surgical strategy but utilizes a two-trajectory approach to promote excellent seizure outcomes^21^. We also performed this strategy in a small cohort of patients (Fig. 4).

The use of advanced minimally invasive MRgLITT was associated with a shorter learning curve than the prolonged learning curve associated with OS involving complex craniotomy techniques. A study reported a significant increase in the adoption of MRgLITT procedures (from 1% in 2012 to 13.2% in 2016), indicating a growing preference for these minimally invasive techniques over traditional OS procedures^22^. A prior study reported a learning curve and increased operator experience of MRgLITT was observed by some authors to decrease the incidence of complications^23^. To address the potential impact of the learning curve, we conducted a subset analysis by dividing patients equally based on the timing of their surgery (early-surgery and late-surgery subsets) and found no statistically significant differences in memory function and seizure freedom rates. Remarkably, we identified a relatively short learning curve for MRgLITT in 15 patients who achieved comparable seizure outcomes. This observation could be attributed to the relatively consistent ablative range for DR-mTLE cases involving the mesial temporal structures. This contrasts with scenarios involving diverse pathologies such as intracranial tumours or cortical dysplasia, in which the size and location may vary considerably.

Minimally invasive MRgLITT offers several advantages over traditional OS in the treatment of DR-mTLE. It allows for real-time monitoring and control of the thermal ablation process using intraoperative MRI, resulting in a smaller incision, reduced risk for infection, shorter duration of hospital stay, shorter recovery durations, and potentially fewer cognitive side effects than those in traditional OS^24^. Since TLE can impact cognitive functioning, including intelligence, attention, memory, and language skills^25^, using scales can help clinicians better understand the cognitive capacities of patients. The Wechsler Intelligence Scale Fourth Edition is commonly used to assess cognitive abilities, such as verbal comprehension, perceptual reasoning, working memory, and processing speed, in patients with TLE^26–29^. In this study, we tracked the trend in memory abilities based on patient-reported subjective perception at the most recent follow-up, which may introduce subjective bias. Additionally, visual deficits should be considered when performing TLE surgery. A recent study proposed a novel quantitative scoring method to assess postoperative visual field deficits after TLE surgery^30^. This method shows potential as a valuable addition to the routine presurgical evaluations before TLE surgery.

### Strengths and limitations

A systematic review of 19 publications reported that MRgLITT had compelling evidence of efficacy; nonetheless, comparisons to surgical resection were limited^31^. Our study presenting evidence comparing MRgLITT and OS in clinical value stands to potentially supplant standard surgery. Seizure control and functional protection are of heightened importance in patients with dominant-side mTLE. Consequently, future prospective randomized trials with high-level evidence and comprehensive evaluations encompassing memory, aphasia, and visual field examinations should provide practical guidelines for neurosurgeons when selecting surgical approaches.

The major limitation of this study was its retrospective nature, which introduced the possibility of selection bias. Despite employing PSM to reduce selection bias, there may still be a residual selection bias from unmeasured or unknown confounding factors that were not accounted for in the absence of randomization. Although not all SMD values were below the threshold of 0.1, the variables matched well between the cohorts after the PSM analysis. It should be noted that the use of a 0.1 threshold for determining covariate balance is somewhat arbitrary, as SMD only compares means between groups and does not consider higher-order moments or covariate interactions. Thus, achieving balance based on SMD does not guarantee similarity in the overall distribution of covariates between the treatment and control groups^32^.

## Conclusions

The findings of this cohort study demonstrate that minimally invasive MRgLITT is associated with similar memory preservation and seizure control as that of traditional OS for DR-mTLE. Furthermore, the results suggest that the MRgLITT procedure may be relatively easy to perform during the learning curve. These findings indicate that MRgLITT is a novel and effective therapy for DR-mTLE.

## Ethical approval

All institutions obtained their respective approvals as per the requirements of their local centre’s requirements. Approval for this study was granted by the Institutional Review Board of Beijing Tiantan Hospital (QX2020-11-02).

## Consent

Written informed consent was obtained from the patient for publication and any accompanying images. A copy of the written consent is available for review by the Editor-in-Chief of this journal on request.

## Sources of funding

The trial was supported by Capitals Funds for Health Improvement and Research (2022-1-1071), National Natural Science Foundation of China (82071457, 82201600, 82201603 and 82271495); National Key R&D Program of China (2021YFC2401201).

## Author contribution

K.Z. had full access to all of the data in the study and takes responsibility for the integrity of the data and the accuracy of the data analysis. J.M. and Z.G. served as co-first authors and contributed equally to this work. Concept and design: K.Z. and W.H. Acquisition, analysis, or interpretation of data: J.M. and Z.G. Drafting of the manuscript: J.M. and W.H. Critical revision of the manuscript for important intellectual content: W.H., C.Z., and X.S. Statistical analysis: J.M. Administrative, technical, or material support: J.Z., W.H., L.S., and Z.Z. Supervision: K.Z.

## Conflicts of interest disclosure

The authors declare no conflict of interest.

## Research registration unique identifying number (UIN)

NCT04569071.

## Guarantor

Jiajie Mo, Wenhan Hu, Kai Zhang.

## Data availability statement

All the other data supporting the findings of this study are available within the article and its Supplementary Information Files, or from the corresponding authors upon reasonable request.

## Provenance and peer review

Not commissioned, externally peer-reviewed.
